# *CDKN2B‐AS1* gene rs4977574 A/G polymorphism and coronary heart disease: A meta‐analysis of 40,979 subjects

**DOI:** 10.1111/jcmm.16849

**Published:** 2021-08-21

**Authors:** Yan‐yan Li, Hui Wang, Yang‐yang Zhang

**Affiliations:** ^1^ Clinical Research Center First Affiliated Hospital of Nanjing Medical University Nanjing China; ^2^ Department of Geriatrics First Affiliated Hospital of Nanjing Medical University Nanjing China; ^3^ Department of Cardiology First Affiliated Hospital of Nanjing Medical University Nanjing China; ^4^ Department of General Practice First Affiliated Hospital of Nanjing Medical University Nanjing China

**Keywords:** *CDKN2B‐AS1*, coronary heart disease, gene, polymorphism, rs4977574

## Abstract

It has been implied that there is a possible relationship between *cyclin‐dependent protein kinase inhibitors antisense RNA 1* (*CDKN2B‐AS1*) gene rs4977574 A/G polymorphism and coronary heart disease (CHD) susceptibility. However, as the research results are discrepant, no distinct consensus on this issue has been reached so far. In order to further elaborate the latent association of the *CDKN2B‐AS1* gene rs4977574 A/G polymorphism and CHD, this present meta‐analysis was conducted. There were 40,979 subjects of 17 individual studies in the present meta‐analysis. The pooled odds ratios (ORs) and their corresponding 95% confidence intervals (CIs) were estimated to determine the association strength. Considering the significant heterogeneity among the individual studies, the random‐effect models were used. In the current meta‐analysis, a significant association between *CDKN2B‐AS1* gene rs4977574 A/G polymorphism and CHD was found under allelic (OR: 1.18, 95% CI: 1.08–1.29, *p* = 4.83×10^−4^), recessive (OR: 1.36, 95% CI: 1.11–1.67, *p* = 0.003), dominant (OR: 0.71, 95% CI: 0.58–0.86, *p* = 6.26×10^−4^), heterozygous (OR:1.210, 95% CI: 1.076–1.360, *p* = 0.001), homozygous (OR: 1.394, 95% CI: 1.163–1.671, *p* = 3.31×10^−4^) and additive (OR: 1.180, 95% CI: 1.075–1.295, *p* = 4.83×10^−4^) genetic models. A more significant association between them was found in the Asian population than that in the whole population under these genetic models (*p* < 0.05). However, no significant association between them was found in the Caucasian population (*p* > 0.05). *CDKN2B‐AS1* gene rs4977574 A/G polymorphism was associated with CHD susceptibility, especially in the Asian population. G allele of *CDKN2B*‐*AS1* gene rs4977574 A/G polymorphism is the risk allele for CHD.

## 1 INTRODUCTION

Cardiovascular disease (CVD) due to atherosclerosis (AS) is the leading cause of death in humans. In Europe, the United States and other developed countries, deaths caused by coronary heart disease (CHD) account for nearly half of the total number of deaths. Approximately 5.7 million Americans, or 2.5% of the population, have CHD, and one in five American men have a chance of acquiring CHD before the age of 60. In accordance with the CVD report in China in 2019, the prevalence of CVD in China is on the rise. The number of CVD patients has reached 330 million, of which CHD is up to 11 million. In 2017, CVD mortality was the highest, higher than that of cancer and other diseases.^1^

The main pathological change of CHD is AS, which is marked by the infiltration of inflammatory cells in the artery wall and the abnormal proliferation of vascular smooth muscle cells (VSMCs), leading to vascular endothelial hyperplasia.^2^ The coronal AS progression is influenced by genetic and environmental factors. Under the coaction of mechanical and chemical injury, vascular endothelial cells are damaged and inflammatory cells stick together, thus promoting the proliferation and migration of vascular endothelial cells and forming AS plaque. Although the aetiology of CHD is incompletely clear, genetic and environmental factors are recognized as the main pathogenesis.^3^, ^4^ Environmental factors include gender, age, obesity, high cholesterol, high blood pressure and unhealthy living habits, which can explain 50%‐60% of the aetiology of CHD. The remaining 40%‐50% of the aetiology of CHD is due to genetic factors.^5^ As a result, the CHD genetic cause factors become the hot spot in pathogenesis research.

Previous research has confirmed that the *cyclin‐dependent kinase inhibitor 2A/2B* (*CDKN2A*/*2B*) gene is associated with the occurrence of coronal AS in the general population.^6^, ^7^ Cyclin‐dependent kinases (CDKs) could catalyse VSMC proliferation and cause atherosclerotic changes. The protein encoded by the *CDKN2A*/*2B* gene could inhibit CDK activities and prevent VSMC cell proliferation and apoptosis. Hence, CDKN2A/2B has the potential protection effect on CHD.

*CDKN2A*/*2B* is an important tumour suppressor gene, belonging to the CDK inhibitor gene family. It is located in the p21.3 band on the short arm of human chromosome 9 and is widely expressed in human cells and tissues as vascular endothelial cells and smooth coronary muscle cells. The *CDKN2A*/*2B* gene contains four exons, 1α, 1β, 2 and 3, encoding two different proteins: P16INK4a (P16) and p14ARF (P14). The human *CDKN2B*‐*AS1* (also known as *ANRIL*), located adjacent to the *CDKN2A*/*2B* gene cluster, is an underlying gene for CHD that encodes an antisense noncoding RNA. The human *CDKN2B*‐*AS1* gene rs4977574 A/G polymorphism is located in the *CDKN2A*/*2B* gene intron, where the adenine (A) nucleotide is being replaced with guanine (G) nucleotide. Although it is a nonprotein coding variant, it might influence the *CDKN2A*/*2B* gene expression directly. Thus, *CDKN2B*‐*AS1* gene rs4977574 A/G polymorphism was reported to be associated with the CHD onset.^8^

Despite many studies on the association of *CDKN2B*‐*AS1* gene rs4977574 A/G polymorphism and CHD have been performed, no clear consensus has been reached. In 2013, Sakalar et al. found that the G allele frequency of *CDKN2B*‐*AS1* gene rs4977574 polymorphism in myocardial infarction patients was significantly higher than that in controls in a Turkish population.^9^ Analogously, in 2020, Hua et al. examined the association between *CDKN2B*‐*AS1* gene rs4977574 polymorphism and CHD in a Chinese population and found that the G allele is the CHD susceptibility site.^10^ On the contrary, in 2007, Samani et al.^11^ reported that the G allele frequency was greatly lower in CHD patients than that in controls in a WTCCC study of a British population.

To verify whether *CDKN2B*‐*AS1* gene rs4977574A/G polymorphism is susceptible to CHD development, a meta‐analysis of 40,979 subjects from 17 separate studies was conducted (Supplements S1).

## 2 MATERIALS AND METHODS

### 2.1 Publication search and inclusion criteria

The terms “*CDKN2A*/*2B*,” “*CDKN2B*‐*AS1*,” “*rs4977574*,” “coronary heart disease,” “myocardial infarction,” “coronary artery disease” and “polymorphism” were used to perform a search. The electronic databases of Wan Fang, PubMed, VIP, Web of Science, Embase and China National Knowledge Infrastructure were retrieved. The selected papers were released between 2007 and 2020. The retrieval process was updated on May 6, 2021.

The collected studies must meet the next inclusion criteria in the current meta‐analysis. The selected studies must evaluate the association of CHD and *CDKN2B*‐*AS1*gene rs4977574 A/G polymorphism. The CHD diagnosis was defined as that the coronary artery stenosis was over 50% in at least one main coronary artery. The coronary artery stenosis was calculated through dual‐source coronary computed tomography or coronary angiography. The selected studies must conform to the Hardy‐Weinberg equilibrium (HWE) in genotype distribution in the control group. The included studies were cohort or case‐control studies that were publicly released.

### 2.2 Data extraction

In accordance with a standardized protocol, the information of individual studies was drawn out by three investigators together. Two of them selected the eligible studies. The third one acted as an intercessor to solve any ambiguous issues between them. Studies that deviated from the inclusion criteria were repeatedly published or did not provide valid data were excluded. If analogical data were presented in different papers by an identical author group, the data could be merely used once in the current meta‐analysis. The definition of controls included the source of controls, that is either hospital‐based or population‐based. The quality of studies was assessed by using the Newcastle‐Ottawa scale scores.

### 2.3 Statistical analyses

In this study, six genetic models as allelic (G allele frequency distribution), recessive (GG vs. AA + AG), dominant (AA vs. AG + GG), heterozygous (AG vs. AA), homozygous (GG vs. AA) and additive (G vs. A) were adopted. The ORs and their 95% CIs were calculated to analyse the association of *CDKN2B*‐*AS1*gene rs4977574 A/G polymorphism and CHD. The heterogeneity among the individual researches was evaluated through the chi‐square‐based Q‐tests.^12^ If there was obvious heterogeneity, the random‐effect model would be applied.^13^ Otherwise, the fixed‐effect model would be utilized.^14^ The significance was set at *p* < 0.05 level in all of the above tests.

The potential publication bias was assessed by using the funnel plot and Egger's linear regression test.^15^ All of the statistical analyses were conducted by utilizing Review Manager 5.3 and Stata 12.0 software.

## 3 RESULTS

### 3.1 Studies and populations

The information was drawn out from 18,430 CHD cases and 22,549 controls (Table 1).^9^, ^10^, ^11^, ^16^, ^17^, ^18^, ^19^, ^20^, ^21^, ^22^, ^23^, ^24^, ^25^, ^26^, ^27^, ^28^, ^29^ Of the 35 obtained literatures after the preliminary search course, 17 met the present meta‐analysis including criteria. Among the eighteen eliminated papers, five of them were of review character. Two of them were against the HWE in the control group.^30^, ^31^ Nine of them lack the indispensable data.^32^, ^33^, ^34^, ^35^, ^36^, ^37^, ^38^, ^39^, ^40^ The rest two excluded papers were not associated with the *CDKN2B*‐*AS1* gene rs4977574 A/G polymorphism or CHD (Supplements S2).

**TABLE 1:** Characteristics of the association between the*CDKN2B*‐*AS1* gene rs4977574 A/G polymorphism and CHD

Author	Year	Region	Ethnicity	CHD			Control			Matching criteria	Source of control	NOS score	Genotyping method	sample size (CHD/control)
				AA	AG	GG	AA	AG	GG					
Sakalar C^9^	2013	Turkey	Caucasian	8	22	14	13	11	4	Age, sex, hypertension, diabetes, hyperlipidaemia family history, ethnicity	HB	6	PCR‐RFLP	44/28
Helgadottir A^16^	2007	Iceland	Caucasian	554	1105	556	1507	2335	964	Ethnicity	PB	6	PCR‐sequencing	2215/4806
Helgadottir A^16^	2007	Philadelphia	Caucasian	103	286	180	130	246	119	Ethnicity	PB	6	PCR‐sequencing	569/495
Helgadottir A^16^	2007	Atlanta	Caucasian	115	274	188	332	597	325	Ethnicity	PB	6	PCR‐sequencing	577/1254
Helgadottir A^16^	2007	Durham	Caucasian	267	549	316	187	383	144	Ethnicity	PB	6	PCR‐sequencing	1132/714
KalpanaB^17^	2019	India	Asian	23	36	31	106	230	100	Hyperlipidaemia, weight, alcohol, ethnicity, smoking, exercise, fruits intake, family history	PB	6	Mass Array Technology	90/436
Hua L^10^	2020	China	Asian	149	297	152	87	122	48	Drinking, TC, TG, LDL‐C, Apo B, Bun, ethnicity	HB	6	Mass Array SNP typing	598/257
SamaniNJ^11^	2007	UK	WTCCC(Caucasian)	605	937	382	698	1435	804	Ethnicity	PB	6	Affymetrix 500K	1924/2937
SamaniNJ^11^	2007	UK	German MI study (Caucasian)	169	452	239	463	826	354	Ethnicity	PB	6	Affymetrix 500K	860/1643
Saade S^18^	2011	Lebanon	Asian	208	685	627	72	195	156	TC, LDL‐C, LogTG, ethnicity, BMI	PB	6	Illumina chips	1520/423
Zheng Y^19^	2016	China	Asian	422	795	343	540	891	320	Age, sex, physical activity, prevalent cholesterolaemia, urban area of residence, individual European admixture proportion	PB	6	PCR‐ASP	1560/1751
Qiao L^20^	2017	China	Asian	43	114	69	26	35	18	Age, BMI,TC,LDL‐C, HDL‐C, ethnicity	HB	6	PCR‐sequencing	226/79
Wang YQ^21^	2014	China	Asian	583	1139	595	777	1325	482	Sex, WHR, alcohol intake, ethnicity	HB	6	PCR‐ASP	2317/2584
Cao XL^22^	2016	China	Asian	117	272	176	152	255	134	Age, sex, smoking, LDL‐C, ApoB, ethnicity	HB	6	PCR‐RFLP, direct sequencing	565/541
Matsuoka R^23^	2015	Japan	Asian	448	898	476	651	1132	501	SBP, ethnicity	PB	6	Luminex bead‐based multiplex assay	1822/2284
Beigi SSH^24^	2015	Iran	Asian	22	44	34	17	44	32	Age, sex, Height, Weight, BMI, TC,TG,FBS, HDL,LDL,SBP, DBP, ethnicity	HB	6	TaqMan SNP genotyping assay	100/93
Tang OS^25^	2017	China	Asian	37	136	116	38	134	166	Age, sex, DBP, ethnicity	HB	6	real‐time PCR	289/338
Lee IT^26^	2014	China	Asian	198	479	248	181	318	135	BMI, HDL,SBP, DBP, ethnicity	HB	6	StepOnePlus Real‐Time PCR	925/634
Qi L^27^	2012	China	Asian	35	64	43	46	94	52	Ethnicity	HB	6	PCR‐RFLP	142/192
Xu JJ^28^	2018	China	Asian	202	253	429	287	182	438	Age, Bleeding, TC, RBC, ethnicity	HB	6	iMLDR	884/907
Temel SG^29^	2019	Turkey	Caucasian	24	33	14	39	76	38	Age, sex, Glucose, TC, ethnicity	HB	6	PCR‐RFLP	71/153

Abbreviations: *CDKN2A/2B*: *Cyclin‐dependent kinase inhibitor 2A/2B*; NOS score: Newcastle‐Ottawa scale scores; PCR‐RFLP: Polymerase chain reaction‐restriction fragment length polymorphism; PCR‐ASP: Polymerase chain reaction‐allelic‐specific primer; iMLDR: improved multiplex ligase detection reaction; TC: total cholesterol; TG: triglyceride; LDL‐C: low‐density lipoprotein cholesterol; Apo B: apolipoproteins B; Bun: blood urea nitrogen; BMI: body mass index; WHR: waist hip rate; SBP: systolic blood pressure; DBP: diastolic blood pressure; RBC: red blood cell.

### 3.2 Pooled analyses

**FIGURE 1:**
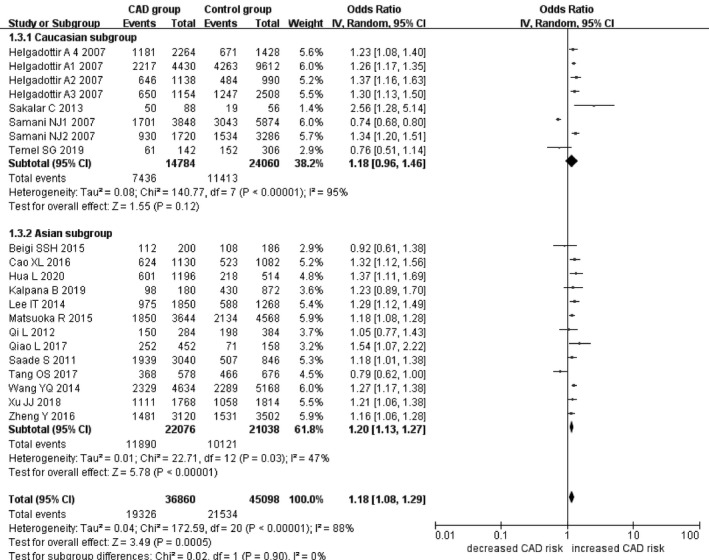
Forest plot of CHD associated with *CDKN2B*‐*AS1* gene rs4977574 A/G polymorphism under an allelic genetic model (distribution of G allele frequency of*CDKN2B*‐*AS1* gene rs4977574 A/G polymorphism)

**FIGURE 2:**
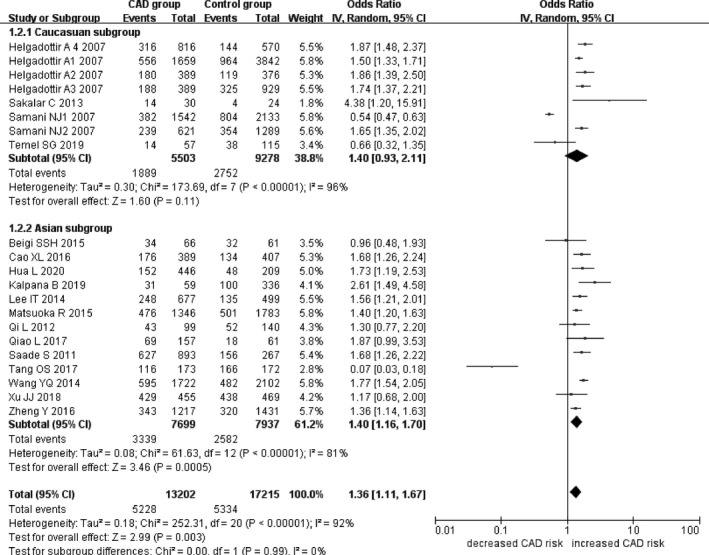
Forest plot of CHD associated with *CDKN2B*‐*AS1* gene rs4977574 A/G polymorphism under a recessive genetic model (GG vs. AG + AA)

**FIGURE 3:**
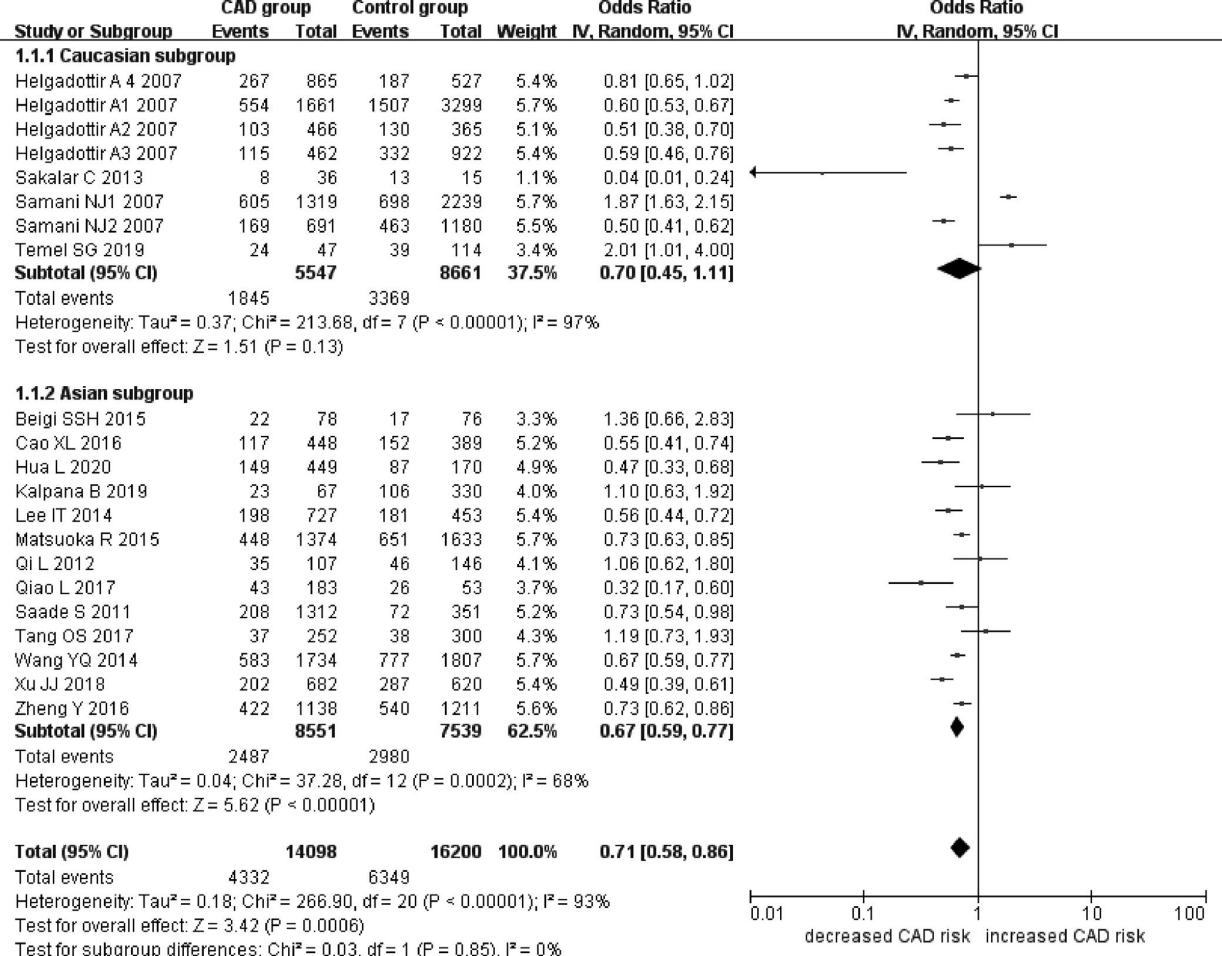
Forest plot of CHD associated with *CDKN2B*‐*AS1* gene rs4977574 A/G polymorphism under a dominant genetic model (AA vs. AG + GG)

**FIGURE 4:**
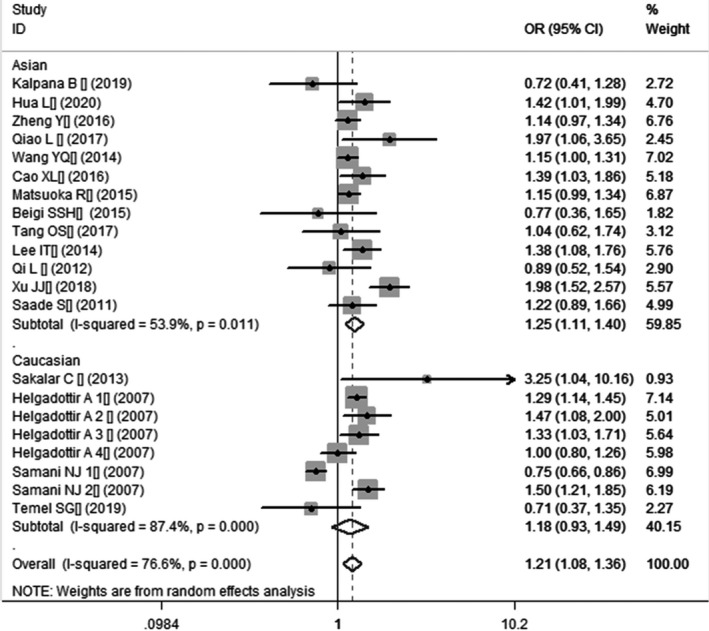
Forest plot of CHD associated with *CDKN2B*‐*AS1* gene rs4977574 A/G polymorphism under a heterozygous genetic model (AG vs. AA)

**FIGURE 5:**
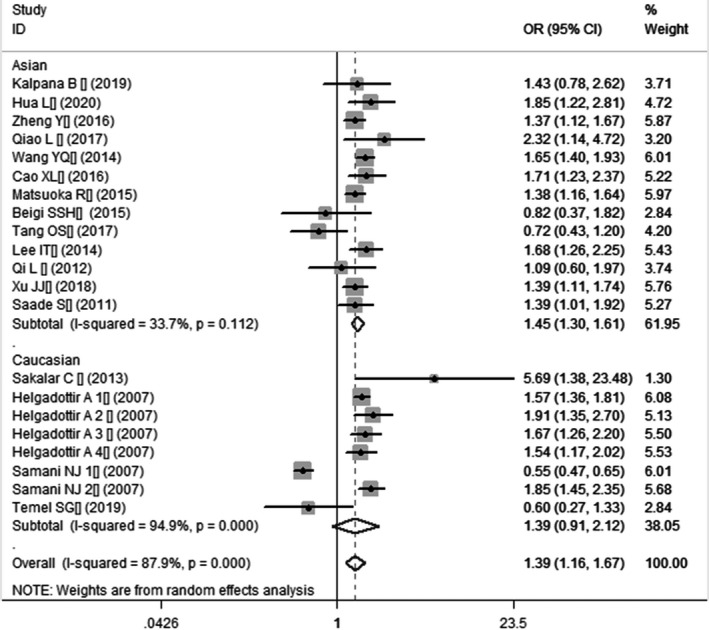
Forest plot of CHD associated with *CDKN2B*‐*AS1* gene rs4977574 A/G polymorphism under a homozygous genetic model (GG vs. AA)

**FIGURE 6:**
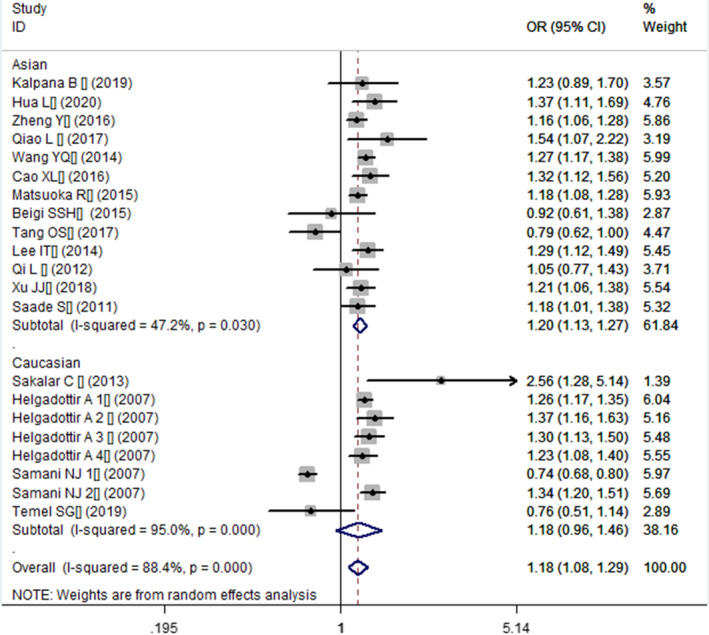
Forest plot of CHD associated with *CDKN2B*‐*AS1* gene rs4977574 A/G polymorphism under an additive genetic model (total G allele vs. total A)

In the current meta‐analysis, a significant association between *CDKN2B*‐*AS1* gene rs4977574 A/G polymorphism and CHD was found under allelic (OR: 1.18, 95% CI: 1.08–1.29, *p* = 4.83×10^−4^), recessive (OR: 1.36, 95% CI: 1.11–1.67, *p* = 0.003), dominant (OR: 0.71, 95% CI: 0.58–0.86, *p* = 6.26×10^−4^), heterozygous (OR:1.210, 95% CI: 1.076–1.360, *p* = 0.001), homozygous (OR: 1.394, 95% CI: 1.163–1.671, *p* = 3.31×10^−4^), and additive (OR: 1.180, 95% CI: 1.075–1.295, *p* = 4.83×10^−4^)genetic models. (Table 2, Figures 1, 2, 3, 4, 5, 6).

**TABLE 2:** Summary of meta‐analysis of association between *CDKN2B*‐*AS1* gene rs4977574 A/G polymorphism and CHD

Genetic model	Pooled OR (95% CI)	*Z* value	*p* Value	Literature number	CHD size	control size	*P*_heterogeneity(_*_I_*^2^%_)_
Allelic genetic model	1.18 (1.08–1.29)	3.49	4.83×10–4*	17	18430	22549	<0.00001 (88%)*
Caucasian subgroup	1.18 (0.96–1.46)	1.55	0.12	4	7392	12030	<0.00001 (95%)*
Asian subgroup	1.20 (1.13–1.27)	5.78	7.47×10^−9^*	13	11038	10519	0.03(47%)*
Recessive genetic model	1.36 (1.11–1.67)	2.99	0.003*	17	18430	22549	<0.00001 (92%)*
Caucasian subgroup	1.40 (0.93–2.11)	1.60	0.11	4	7392	12030	<0.00001(96%)*
Asian subgroup	1.40 (1.16–1.70)	3.46	5.40×10^−4^*	13	11038	10519	<0.00001(81%)*
Dominant genetic model	0.71 (0.58–0.86)	3.42	6.26×10^−4^*	17	18430	22549	<0.00001 (93%)*
Caucasian subgroup	0.70 (0.45–1.11)	1.51	0.13	4	7392	12030	<0.00001 (97%)*
Asian subgroup	0.67 (0.59–0.77)	5.62	1.91×10^−8^*	13	11038	10519	0.0002(68%)*
Heterozygous genetic model	1.210 (1.076–1.360)	3.19	0.001*	17	18430	22549	<0.00001 (76.6%)*
Caucasian subgroup	1.175 (0.928–1.489)	1.34	0.181	4	7392	12030	<0.00001 (87.4%)*
Asian subgroup	1.247 (1.109–1.401)	3.70	2.16×10^−4^*	13	11038	10519	0.011(53.9%)*
Homozygous genetic model	1.394 (1.163–1.671)	3.59	3.31×10^−4^*	17	18430	22549	<0.00001 (87.9%)*
Caucasian subgroup	1.393 (0.913–2.124)	1.54	0.124	4	7392	12030	<0.00001 (94.9%)*
Asian subgroup	1.451 (1.304–1.613)	6.86	6.89×10^−12^*	13	11038	10519	0.112(33.7%)
Additive genetic model	1.180 (1.075–1.295)	3.49	4.83×10^−4^*	17	18430	22549	<0.00001 (88.4%)*
Caucasian subgroup	1.182 (0.956–1.461)	1.55	0.122	4	7392	12030	<0.00001 (95.0%)*
Asian subgroup	1.199 (1.127–1.274)	5.78	7.47×10^−9^*	13	11038	10519	0.03 (47.3%)*

Abbreviations: CHD: coronary heart disease; CI: confidence interval; OR: odds ratio; CHD size: the total number of CHD cases; control size: the total number of control group; Allelic genetic model: G allele distribution frequency; Dominant genetic model: AAvs.AG + GG; Recessive:GGvs.AA + AG; Heterozygous genetic model: AG vs. AA; Homozygous genetic model: GG vs. AA; Additive genetic model: total G allele vs. total A.

*p ≤ 0.05.

In the subsequent subgroups analysis stratified by the ethnicity, the relationship between the *CDKN2B*‐*AS1* gene rs4977574 A/G polymorphism and CHD was found more prominent in the Asian subgroup than that in the whole population. In the Asian subgroup, there was a significant relationship under allelic (OR: 1.20, 95% CI: 1.13–1.27, *p* = 7.47×10^−9^), recessive (OR: 1.40, 95% CI: 1.16–1.70, *p* = 5.40×10^−4^), dominant (OR: 0.67, 95% CI: 0.59–0.77, *p* = 1.91×10^−8^), heterozygous (OR: 1.247, 95% CI: 1.109–1.401, *p* = 2.16×10^−4^), homozygous (OR: 1.451, 95% CI: 1.304–1.613, *p* = 6.89×10^−12^) and additive (OR: 1.199, 95% CI: 1.127–1.274, *p* = 7.92×10^−9^) genetic models. In the Caucasian subgroup, no significant association was detected between *CDKN2B*‐*AS1* gene rs4977574 A/G polymorphism and CHD under allelic (OR: 1.18, 95% CI: 0.96–1.46, *p* = 0.12), recessive (OR: 1.40, 95% CI: 0.93–2.11, *p* = 0.11), dominant (OR: 0.70, 95% CI: 0.45–1.11, *p* = 0.13), heterozygous (OR: 1.175, 95% CI: 0.928–1.489, *p* = 0.181), homozygous (OR: 1.393, 95% CI: 0.913–2.124, *p* = 0.124) and additive (OR: 1.182, 95% CI: 0.956–1.461, *p* = 0.122) genetic models.

In the overall population, the heterogeneity among the individual studies was detected significant under all of the genetic models (*p* < 0.05). However, in the subgroup analysis stratified by the ethnicity, the heterogeneity was decreased under these genetic models in the Asian subgroup. Among them, the heterogeneity even did not exist any longer under the homozygous genetic models (*p* > 0.05). However, in the Caucasian subgroup, the heterogeneity remained significant and the *I* ^2^(%) was still more than 87.4%. Hence, it was shown that the heterogeneity might be originated from the ethnicity.

### 3.3 Bias diagnostics

**FIGURE 7:**
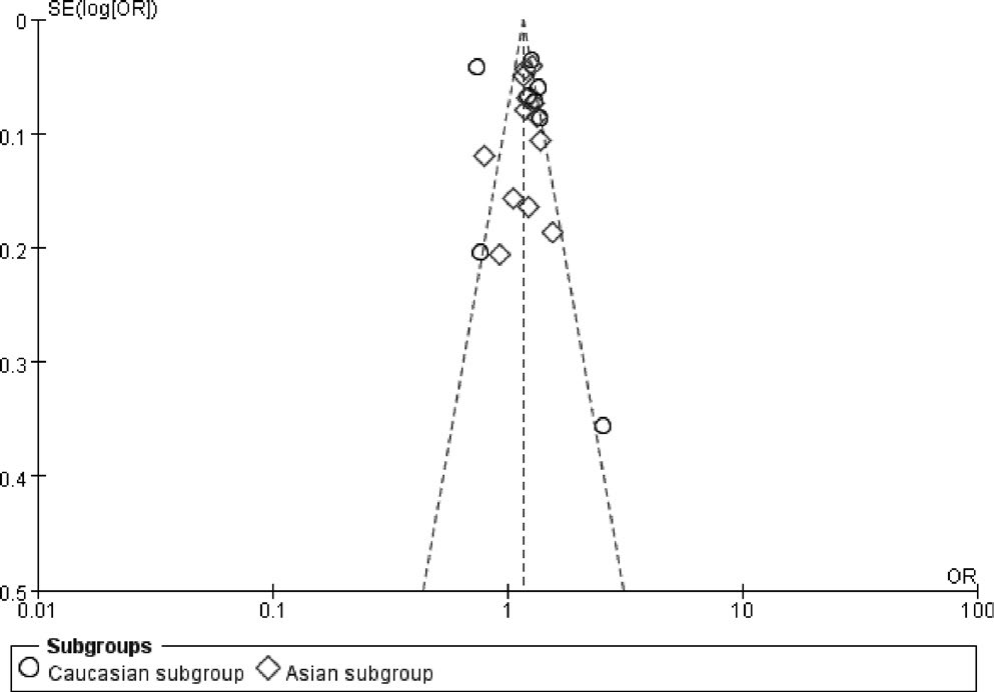
The funnel plot for studies of the association of CHDand*CDKN2B*‐*AS1* gene rs4977574 A/G polymorphism under an allelic genetic model (distribution of G allele frequency of *CDKN2B*‐*AS1* gene rs4977574 A/G polymorphism). The horizontal and vertical axis correspond to the OR and *SE* (log[OR]). OR: odds ratio; *SE*: standard error

**FIGURE 8:**
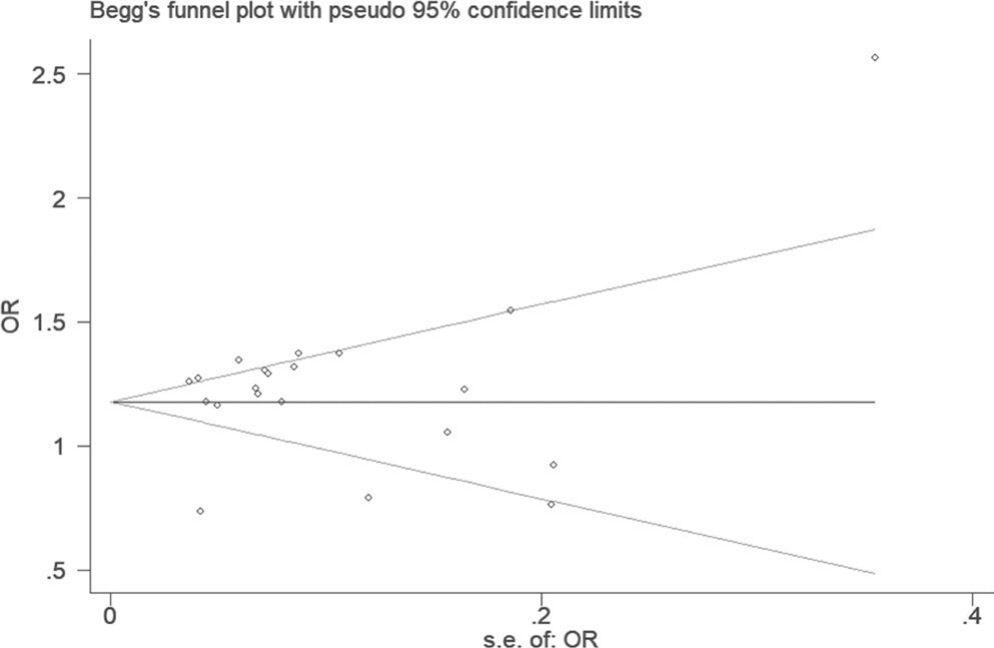
The Begg's funnel plot for studies of the association of CHD and *CDKN2B*‐*AS1* gene rs4977574 A/G polymorphism under an additive genetic model (total G allele vs. total A). The vertical and horizontal axis correspond to the OR and *SE* of OR. OR: odds ratio; *SE*: standard error

In the funnel plot under the allelic genetic model, no publication bias was visualized (Figure 7). Moreover, no significant difference was found by utilizing Egger's test under the additive genetic model. It was suggested that there was no publication bias in the current meta‐analysis under the additive genetic model (*T* =0.79, *p* = 0.448) (Figure 8).

### 3.4 Sensitivity analysis

**FIGURE 9:**
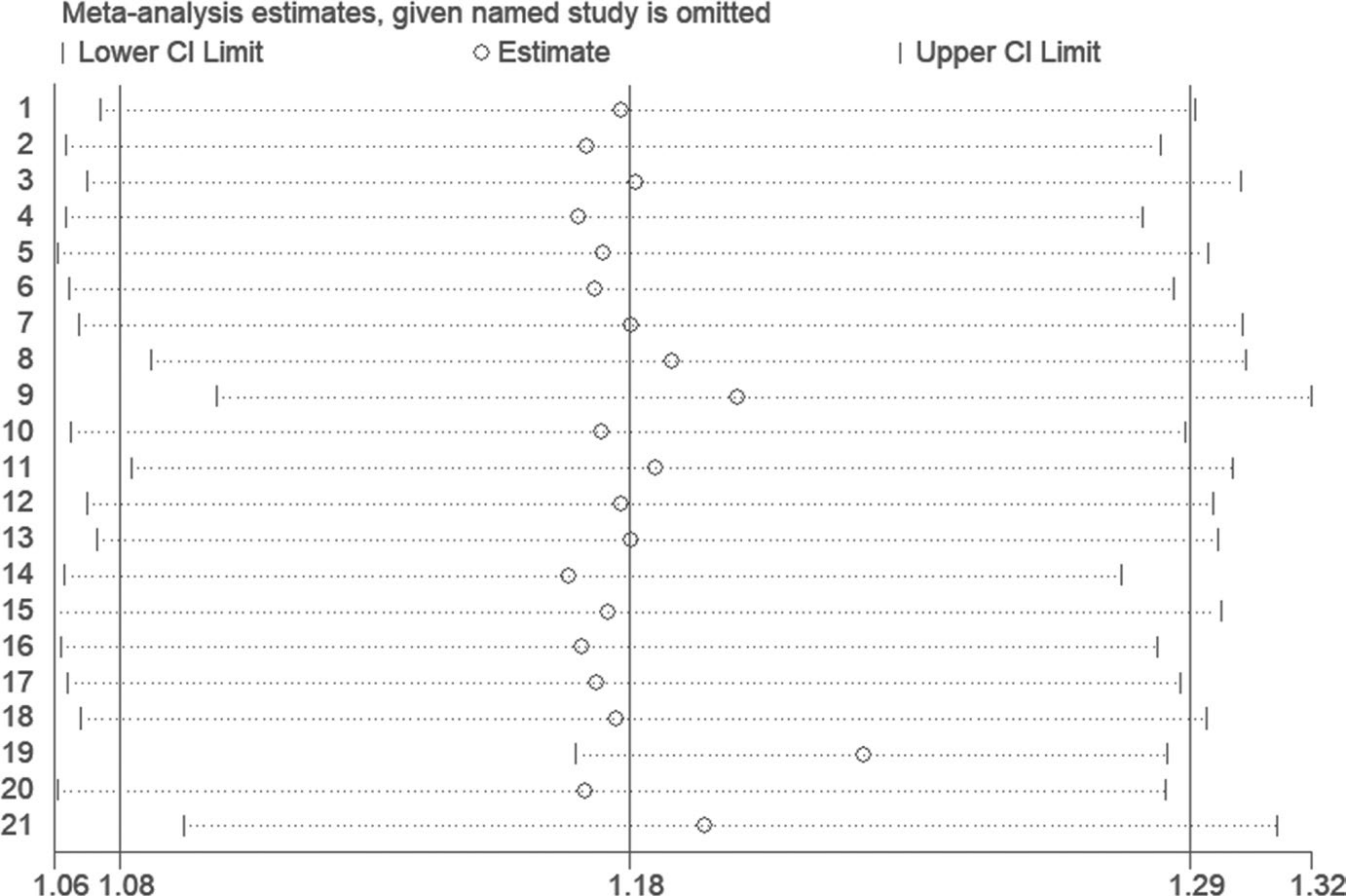
The sensitivity analysis on the association of CHD and *CDKN2B*‐*AS1* gene rs4977574 A/G polymorphism under an additive genetic model (total G allele vs. total A)

Sensitivity analysis was performed under the additive genetic model. After any individual study was removed from the current meta‐analysis, the primary analysis result was unaffected. This result was comparatively steady (Figure 9).

## 4 DISCUSSION

In this meta‐analysis, a remarkable association between *CDKN2B*‐*AS1* gene rs4977574 A/G polymorphism and CHD was observed in the entire population under the allelic (OR: 1.18), recessive (OR: 1.36), dominant (OR: 0.71), heterozygous (OR: 1.210), homozygous (OR: 1.394) and additive (OR: 1.180) genetic models. In addition, the G allele of *CDKN2A*/*2B* gene rs4977574 A/G polymorphism was susceptible to CHD development. Given the evident heterogeneity among individual studies (*p* < 0.00001), a subgroup analysis by ethnicity was performed. In the subgroup analysis, the relationship between *CDKN2B*‐*AS1* gene rs4977574 A/G polymorphism and CHD was more prominent in the Asian subgroup than in the Caucasian subgroup, while the heterogeneity in the Asian subgroup was lower than that in the Caucasian subgroup. The heterogeneity source might be associated with the different ethnicities.

Recent studies have found that the *CDKN2A*/*2B* gene is only approximately 100 kb apart from the chromosome 9p21 risk gene, which is generally expressed in many tissue and cell types. The two kinds of proteins encoded by the *CDKN2A*/*2B* gene are the INK4 family member p16 (p16INK4a) and p14arf, which act as tumour suppressors by regulating the cell cycle. p16 inhibits CDK4 and CDK6, thus activates the retinoblastoma family protein, and then stops the G1 phase of cell transition to S phase. The function of p14ARF (known asp19ARF in mice) is activating p53 tumour suppressor 2. Somatic mutations in CDKN2A are common in human cancers, and CDKN2A is estimated to be the second most frequently inactivated gene in cancer after p53. One of the homologous genes in *CDKN2A* is *CDKN2B*, and they have the same biological function.^41^

CDKN2A/2B mainly inhibits VSMC proliferation in the cell cycle regulation, which is closely related to the pathogenesis of AS of CHD.^42^ The possible mechanisms of *CDKN2B*‐*AS1* gene rs4977574 A/G polymorphism affecting the AS formation remain unclear. It might be closely related to the direct influence of intron variation on *CDKN2A*/*2B* gene expression.^8^ The product of this *CDKN2B*‐*AS1* gene is a functional RNA molecule that interacts with polycomb repressive complexes 1 and 2 resulting in *CDKN2A*/*2B’*epigenetic silencing.^43^ *CDKN2B*‐*AS1* gene rs4977574 A/G mutation may induce overexpression of *CDKN2B*‐*AS1* transcript, thereby inhibiting the expression of CDKN2A/2B. Related studies have shown that the *CDKN2B*‐*AS1* gene expression level is increased in peripheral blood mononuclear macrophages containing the G allele of rs4977574 polymorphism, which can lead to biological behaviours, such as increased cell proliferation and adhesion, and AS.^44^ Ding et al.^45^ reported that CHD patients had lower expression of CDKN2A and CDKN2B than healthy people, which suggested that the decreased CDKN2A and CDKN2B level was associated with the CHD pathogenesis. Previous studies have shown that the transcription level of *CDKN2B*‐*AS1* is strongly correlated with the severity of AS. *CDKN2B*‐*AS1* knockdown in vascular smooth muscle changes the gene expression, which is involved in extracellular matrix remodelling. *CDKN2B*‐*AS1* plays a pivotal role in the reconstitution of vascular structure and function alterations.^46^

Qiao et al. performed a correlation analysis of rs4977574 polymorphism and biomarker. They found that the risk of high HbA1c in GG + GA genotype subjects was significantly increased (OR = 2.08, 95%CI: 1.11–3.89, *p* = 0.022).^20^ They speculated that rs4977574 polymorphism might be involved in the regulation of glucose metabolism. The G allele of rs4977574 polymorphism might be associated with impaired pancreatic beta cell function and decreased insulin secretion. CDK4 is an effective beta cell proliferative regulator of the pancreas. Abnormal *CDKN2A*/*2B* gene expression could lead to pancreatic hypoplasia and diabetes.^47^ Type 2 diabetes mellitus is a disease with an equal risk to that of CHD. Abnormal blood glucose metabolism increases the risk of early CHD. Some studies have found that, under the influence of risky SNP loci, certain transcription products of the *CDKN2B*‐*AS1* gene can regulate the expression of *ADIPOR1*, *VAMP3* and *C11ORF10*; the above‐mentioned genes play an important role in the regulation of metabolic activities, such as blood glucose and lipid.^48^ Therefore, rs4977574 might be involved in the occurrence and development of early‐onset CHD by affecting blood glucose and lipid metabolism through related signalling pathways.^20^

Similar meta‐analyses on *CDKN2B*‐*AS1* gene rs4977574 polymorphism and CHD have been conducted in the past. In 2014, Huang et al.^30^ performed a case‐control study and a meta‐analysis on the association between them. They found a strong association between rs4977574 and CHD risk. However, their case‐control study was against HWE. Hence, their conclusion was not as reliable as that deduced from the current meta‐analysis. In 2018, Xu et al.^49^ performed a meta‐analysis and found that rs4977574 polymorphism was associated with the CHD risk in Asian population. The G allele might contribute to a high risk of CHD. Although they obtained a similar conclusion to that of the current meta‐analysis, they limited the studies in the Asian population. They also included studies against HWE. In the current meta‐analysis, the research scope was expanded, which included Asian and Caucasian studies. Several new studies published after 2018 were also included. In 2020, Zhang et al. performed a meta‐analysis on this subject and obtained a similar conclusion.^50^ Nevertheless, they also included studies deviating from HWE. In addition, they mixed Asian and Caucasian studies and did not divide them into two subgroups. The individual studies included in their meta‐analysis were not as complete as those in the current meta‐analysis. In the current meta‐analysis, a subgroup analysis stratified by ethnicity was performed. Yuan et al. also performed a meta‐analysis on rs4977574 polymorphism and CHD in 2020 and obtained similar results.^51^ However, in their meta‐analysis, individual studies deviating from HWE were included. Although they divided the participating subjects into three subgroups, namely West Asian, East Asian, and Caucasian, the included individual studies were not complete yet. Therefore, the current meta‐analysis is much more comprehensive and convincing than the previous ones.

Nonetheless, this research still has some shortcomings. Multiple large‐scale studies remain insufficient to determine the exact association of *CDKN2B*‐*AS1* gene rs4977574 polymorphism and CHD. Environmental factors, such as smoking, lack of exercise, excessive eating and obesity, also have a significant effect on the CHD process. The microeffects of other genes involved in CHD have yet to be adequately clarified (eg *PCSK9* gene E670G polymorphism and *TGF*‐*β1* gene‐509C/T polymorphism).^52^, ^53^ The *CDKN2B*‐*AS1* gene rs1333040C>T, rs1333042 A>G and rs10757274 A>G variants might also affect the pathogenesis of CHD patients.^50^

In sum, *CDKN2B*‐*AS1* gene rs4977574 polymorphism was evidently relevant to CHD risk, particularly in the Asian population. The G allele of *CDKN2B*‐*AS1* gene rs4977574 polymorphism might confer the CHD risk to the population. Additional large‐scale studies on the association of *CDKN2B*‐*AS1* gene rs4977574 polymorphism and CHD should be conducted to verify this viewpoint in the near future.

## CONFLICT OF INTEREST

The authors have no other conflicts of interest to disclose.

## AUTHOR CONTRIBUTIONS

**Yan‐yan Li:** Conceptualization (lead); Data curation (lead); Formal analysis (lead); Funding acquisition (lead); Investigation (lead); Methodology (lead); Project administration (lead); Resources (lead); Software (lead); Supervision (lead); Validation (lead); Visualization (lead); Writing‐original draft (lead); Writing‐review & editing (lead). **Hui Wang:** Conceptualization (equal); Data curation (equal); Formal analysis (equal); Funding acquisition (equal); Investigation (equal); Methodology (equal); Project administration (equal); Resources (supporting); Software (supporting); Supervision (supporting); Validation (supporting); Visualization (supporting); Writing‐original draft (supporting); Writing‐review & editing (supporting). **Yang‐yang Zhang:** Conceptualization (equal); Data curation (equal); Formal analysis (supporting); Funding acquisition (supporting); Investigation (supporting); Methodology (equal); Project administration (equal); Resources (equal); Software (supporting); Supervision (supporting); Validation (supporting); Visualization (supporting); Writing‐original draft (supporting); Writing‐review & editing (supporting).

## Supporting information

Supplementary Material

Supplementary Material

## Data Availability

The data that support the findings of this study are available from the corresponding author upon reasonable request.

## References

[1] Chinese Cardiovascular health and Disease Report Writing Group . Summary of China’s cardiovascular health and disease report 2019. Chin Circ J. 2020;35:833‐854.

[2] Basatemur GL, Jørgensen HF, Clarke MCH, Bennett MR, Mallat Z. Vascular smooth muscle cells in atherosclerosis. Nat Rev Cardiol. 2019;16:727‐744. 31243391 10.1038/s41569-019-0227-9

[3] Cheng YC, Sheen JM, Hu WL, Hung YC. Polyphenols and oxidative stress in atherosclerosis‐related ischemic heart disease and stroke. Oxid Med Cell Longev. 2017;2017:8526438. 29317985 10.1155/2017/8526438 PMC5727797

[4] Roberts R, Campillo A, Schmitt M. Prediction and management of CAD risk based on genetic stratification. Trends Cardiovasc Med. 2020;30:328‐334. 31543237 10.1016/j.tcm.2019.08.006

[5] Leander K, Hallqvist J, Reuterwall C, Ahlbom A, de Faire U. Family history of coronary heart disease, a strong risk factor for myocardial infarction interacting with other cardiovascular risk factors: results from the Stockholm Heart Epidemiology Program (SHEEP). Epidemiology. 2001;12:215‐221. 11246583 10.1097/00001648-200103000-00014

[6] Abe J, Zhou W, Taguchi J, et al. Suppression of neointimal smooth muscle cell accumulation in vivo by antisense cdc2 and cdk2 oligonucleotides in rat carotid artery. BiochemBiophysRes Commun. 1994;198:16‐24. 10.1006/bbrc.1994.1003 8292019

[7] Ihling C, Technau K, Gross V, Schulte‐Mönting J, Zeiher AM, Schaefer HE. Concordant upregulation of type II‐TGF‐beta‐receptor, the cyclin‐dependent kinases inhibitor P27Kip1 and cyclin E in human atherosclerotic tissue: implications for lesion cellularity. Atherosclerosis. 1999;144:7‐14. 10381272 10.1016/s0021-9150(99)00032-5

[8] Nott A, Meislin SH, Moore MJ. A quantitative analysis of intron effects on mammalian gene expression. RNA. 2003;9:607‐617. 12702819 10.1261/rna.5250403 PMC1370426

[9] Sakalar C, Gurbuz E, Kalay N, Kaya MG. Higher frequency of rs4977574 (the G Allele) on chromosome 9p21.3 in patients with myocardial infarction as revealed by PCR‐RFLP analysis. Tohoku J ExpMed. 2013;230:171‐176. 10.1620/tjem.230.171 23856978

[10] Hua L, Yuan JX, He S, et al. Analysis on the polymorphisms of site RS4977574, andRS1333045 in region 9p21 and the susceptibility of coronary heart disease in Chinese population. BMC Med Genet. 2020;21:36. 32066403 10.1186/s12881-020-0965-x PMC7026955

[11] Samani NJ, Erdmann J, Hall AS, et al. Genomewide association analysis of coronary artery disease. N Engl J Med. 2007;357:443‐453. 17634449 10.1056/NEJMoa072366 PMC2719290

[12] Cochran WG. The effectiveness of adjustment by subclassification in removing bias in observational studies. Biometrics. 1968;24:295‐313. 5683871

[13] Mantel N, Haenszel W. Statistical aspects of the analysis of data from retrospective studies of disease. J Natl Cancer Inst. 1959;22:719‐748. 13655060

[14] DerSimonian R, Laird N. Meta‐analysis in clinical trials. Control Clin Trials. 1986;7:177‐188. 3802833 10.1016/0197-2456(86)90046-2

[15] Egger M, Davey Smith G, Schneider M, Minder C. Bias in meta‐analysis detected by a simple, graphical test. Br Med J. 1997;315:629‐634. 9310563 10.1136/bmj.315.7109.629 PMC2127453

[16] Helgadottir A, Thorleifsson G, Manolescu A, et al. A common variant on chromosome 9p21 affects the risk of myocardial infarction. Science. 2007;316:1491‐1493. 17478679 10.1126/science.1142842

[17] Kalpana B, Murthy DK, Balakrishna N, Aiyengar MT. Genetic variants of chromosome 9p21.3 region associated with coronary artery disease and premature coronary artery disease in an Asian Indian population. Indian Heart J. 2019;71;263‐271. 31543200 10.1016/j.ihj.2019.04.005 PMC6796635

[18] Saade S, Cazier JB, Ghassibe‐Sabbagh M, et al. Large scale association analysis identifies three susceptibility loci for coronary artery disease. PLoS One. 2011;6:e29427. 22216278 10.1371/journal.pone.0029427 PMC3246490

[19] Zheng Y, Li Y, Huang T, Cheng HL, Campos H, Qi L. Sugar‐sweetened beverage intake, chromosome 9p21 variants, and risk of myocardial infarction in Hispanics. Am J ClinNutr. 2016;103:1179‐1184. 10.3945/ajcn.115.107177 PMC4807696 26961926

[20] Qiao L, Wen XY, Dou KF, et al. Correlation study between CDKN2B‐AS1 gene polymorphism and female premature coronary artery disease occurrence. Chinese Circ J. 2017;32:1154‐1157.

[21] Wang Y, Wang L, Liu X, et al. Genetic variants associated with myocardial infarction and the risk factors in Chinese population. PLoS One. 2014;9:e86332. 24475106 10.1371/journal.pone.0086332 PMC3903528

[22] Cao XL, Yin RX, Huang F, Wu JZ, Chen WX. Chromosome 9p21 and ABCA1 genetic variants and their interactions on coronary heart disease and ischemic stroke in a Chinese Han population. Int J Mol Sci. 2016;17:586. 27096864 10.3390/ijms17040586 PMC4849041

[23] Matsuoka R, Abe S, Tokoro F, et al. Association of six genetic variants with myocardial infarction. Int J Mol Med. 2015;35:1451‐1459. 25738804 10.3892/ijmm.2015.2115

[24] Beigi SSH, Ghaderian SMH, Doosti A. Investigation of the association between rs4977574 A > G polymorphism in ANRIL gene and coronary artery disease in Iranian population. IntCardiovasc Res J. 2015;9:139‐144.

[25] Tang O, Lv J, Cheng Y, Qin F. The correlation between 9p21 chromosome rs4977574 polymorphism genotypes and the development of coronary artery heart disease. Cardiovasc Toxicol. 2017;17:185‐189. 27240780 10.1007/s12012-016-9372-0

[26] Lee IT, Goodarzi MO, Lee WJ, et al. The chromosome 9p21 variant not predicting long‐term cardiovascular mortality in Chinese with established coronary artery disease: an eleven‐year follow‐up study. Biomed Res Int. 2014;2014:1‐8. 10.1155/2014/626907 PMC3996981 24804228

[27] Qi L, Li JM, Sun H, et al. Association between gene polymorphisms and myocardial infarction in Han Chinese of Yunnan province. Zhonghua Yi Xue Yi Chuan Xue Za Zhi. 2012;29:413‐419. 22875497 10.3760/cma.j.issn.1003-9406.2012.04.008

[28] Xu JJ, Jiang L, Xu LJ, et al. Association of CDKN2B‐AS1 polymorphisms with premature triple‐vessel coronary disease and their sex specificity in the Chinese population. Biomed Environ Sci. 2018;31:787‐796. 30558699 10.3967/bes2018.106

[29] Temel ŞG, Ergören MÇ. The association between the chromosome 9p21 CDKN2B‐AS1gene variants and the lipid metabolism: a pre‐diagnostic biomarker for coronary artery disease. Anatol J Cardiol. 2019;21:31‐38. 30587704 10.14744/AnatolJCardiol.2018.90907 PMC6382903

[30] Huang Y, Ye H, Hong Q, et al. Association of CDKN2BAS polymorphism rs4977574 with coronary heart disease: a case‐control study and a meta‐analysis. Int J MolSci. 2014;15:17478‐17492. 10.3390/ijms151017478 PMC4227174 25268619

[31] Wang W, Yin RX. The relevant study between single nucleotide polymerphisms and susceptibility and clinical characteristics of acute myocardial infarction. PhD thesis, Guangxi Medical University; 2016, p. 63.

[32] Shanker J, Arvind P, Jambunathan S, Nair J, Kakkar V. Genetic analysis of the 9p21.3 CAD risk locus in Asian Indians. ThrombHaemost. 2014;111:960‐969. 10.1160/TH13-08-0706 24452806

[33] Lee JY, Lee BS, Shin DJ, et al. A genome‐wide association study of a coronary artery disease risk variant. J Hum Genet. 2013;58:120‐126. 23364394 10.1038/jhg.2012.124

[34] AbdulAzeez S, Al‐Nafie AN, Al‐Shehri A, et al. Intronic polymorphisms in the CDKN2B‐AS1 gene are strongly associated with the risk of myocardial infarction and coronary artery disease in the Saudi population. Int J Mol Sci. 2016;17:395. 26999117 10.3390/ijms17030395 PMC4813250

[35] Erdmann J, Grosshennig A, Braund PS, et al. New susceptibility locus for coronary artery disease on chromosome 3q22.3. Nat Genet. 2009;41:280‐282. 19198612 10.1038/ng.307 PMC2695543

[36] Myocardial Infarction Genetics Consortium , Kathiresan S, Voight BF, Purcell S, et al. Genome‐wide association of early‐onset myocardial infarction with single nucleotide polymorphisms and copy number variants. Nat Gene. 2009;41:334‐341. 10.1038/ng.327 PMC2681011 19198609

[37] Davies RW, Dandona S, Stewart AF, et al. Improved prediction of cardiovascular disease based on a panel of single nucleotide polymorphisms identified through genome‐wide association studies. CircCardiovasc Genet. 2010;3:468‐474. 10.1161/CIRCGENETICS.110.946269 PMC3035486 20729558

[38] Qi L, Parast L, Cai T, et al. Genetic susceptibility to coronary heart disease in type 2 diabetes: 3 independent studies. J Am CollCardiol. 2011;58:2675‐2682. 10.1016/j.jacc.2011.08.054 PMC3240896 22152955

[39] Coronary Artery Disease (C4D) Genetics Consortium . A genome‐wide association study in Europeans and South Asians identifies five new loci for coronary artery disease. Nat Genet. 2011;43:339‐344. 21378988 10.1038/ng.782

[40] Erdmann J, Willenborg C, Nahrstaedt J, et al. Genome‐wide association study identifies a new locus for coronary artery disease on chromosome 10p11.23. Eur Heart J. 2011;32:158‐168. 21088011 10.1093/eurheartj/ehq405

[41] Liu MT, Liu JY, Su J. CDKN2A gene in melanoma. Zhonghua Bing Li XueZaZhi. 2019;48:909‐912. 10.3760/cma.j.issn.0529-5807.2019.11.019 31775447

[42] Karvanen J, Silander K, Kee F, et al. The impact of newly identified loci on coronary heart disease, stroke and total mortality in the MORGAM prospective cohorts. Genet Epidemiol. 2009;33:237‐246. 18979498 10.1002/gepi.20374 PMC2696097

[43] Burd CE, Jeck WR, Liu Y, et al. Expression of linear and novel circular forms of an INK4/ARF‐associated non‐coding RNA correlates with atherosclerosis risk. PLoS Genet. 2010;6:e1001233. 21151960 10.1371/journal.pgen.1001233 PMC2996334

[44] Holdt LM, Hoffmann S, Sass K, et al. Alu elements in ANRIL noncoding RNA at chromosome 9p21 modulate atherogenic cell functions through trans‐regulation of gene networks. PLoS Genet. 2013;9:e1003588. 23861667 10.1371/journal.pgen.1003588 PMC3701717

[45] Ding XF, Hu SJ. Correlation of CDKN2A, CDKN2B, MEF2A and VEGF gene expressions and coronary heart disease. J China Med Univ. 2016;45:550‐553.

[46] Jarinova O, Stewart AF, Roberts R, et al. Functional analysis of the chromosome9p21.3 coronary artery disease risk locus. ArteriosclerThrombVascBiol. 2009;29:1671‐1677. 10.1161/ATVBAHA.109.189522 19592466

[47] Zhang Y, Duan HJ. Expression of cyclin D1, CDK4 and p16 in renalt issues at early stage of experimental diabetes. Master's thesis, Hebei Medical University; 2013, p. 25.

[48] Bochenek G, Häsler R, El Mokhtari NE, et al. The large non‐coding RNA ANRIL, which is associated with atherosclerosis, periodontitis and several forms of cancer, regulates ADIPOR1, VAMP3 and C11ORF10. Hum Mol Genet. 2013;22:4516‐4527. 23813974 10.1093/hmg/ddt299

[49] Xu B, Fang Z, He S, Wang J, Yang X. ANRIL polymorphism rs4977574 is associated with increased risk of coronary artery disease in Asian populations: a meta‐analysis of 12,005 subjects. Medicine (Baltimore). 2018;97:e12641. 30278588 10.1097/MD.0000000000012641 PMC6181537

[50] Zhang YN, Qiang B, Fu LJ. Association of ANRIL polymorphisms with coronary artery disease. Medicine. 2020;99(42):e22569. 33080691 10.1097/MD.0000000000022569 PMC7571950

[51] Yuan W, Zhang W, Zhang W, et al. New findings in the roles of cyclin‐dependent kinase inhibitors 2B antisense RNA 1(CDKN2B‐AS1) rs1333049 G/C and rs4977574 A/G variants on the risk to coronary heart disease. Bioengineered. 2020;11:1084‐1098. 33054494 10.1080/21655979.2020.1827892 PMC8291866

[52] Li YY, Wang H, Yang XX, Geng HY, Gong G, Lu XZ. *PCSK9* gene E670Gpolymorphism and coronary artery disease: an updated meta‐analysis of 5,484subjects. Front Cardiovasc Med. 2020;7:582865. 10.3389/fcvm.2020.582865 PMC7683799 33244470

[53] Li YY, Zhou YH, Gong G, Geng HY, Yang XX. *TGF‐β1* gene ‐509C/T polymorphism and coronary artery disease: an updated meta‐analysis involving 11,701 subjects. Front Physiol. 2017;8:108. 28280469 10.3389/fphys.2017.00108 PMC5322195

