# Supplementary material for: CDKN2B‐AS1 gene rs4977574 A/G polymorphism and coronary heart disease: A meta‐analysis of 40,979 subjects
Source: J Cell Mol Med. 2021 Aug 21;25(18):8877–89. doi: 10.1111/jcmm.16849 (PMC8435436; doi:10.1111/jcmm.16849)
Supplement: Supplementary file 2 — Supplementary Material [file JCMM-25-8877-s002.docx]

**PRISMA 2009 Flow Diagram**


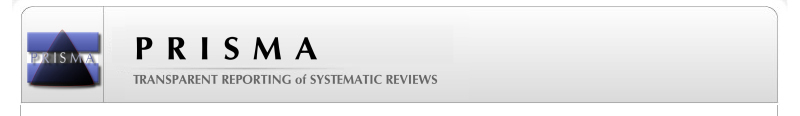


Records excluded for lacking the indispensable data
(n = 9 )

Records excluded for no association with *CDKN2B-AS1* gene rs4977574 A/G polymorphism or CHD

(n =2)

Full-text articles excluded for deviation from HWE (n =2)

Records excluded for review characteristic
(n =5 )

Studies included in qualitative synthesis
(n =17)

Full-text articles assessed for eligibility
(n =19)

Records screened
(n =21 )

Records after duplicates removed
(n =26)

Additional records identified through other sources
(n =0 )

## Identification

## Eligibility

## Included

## Screening

Records identified through database searching
(n =35 )
